# Waist circumference is associated with major adverse cardiovascular events in male but not female patients with type-2 diabetes mellitus

**DOI:** 10.1186/s12933-020-01007-6

**Published:** 2020-03-25

**Authors:** Zhenhua Xing, Zhenyu Peng, Xiaopu Wang, Zhaowei Zhu, Junyu Pei, Xinqun Hu, Xiangping Chai

**Affiliations:** 1 Department of Emergency Medicine, Second Xiangya Hospital, Central South University, Changsha, 410011 China; 2 Emergency Medicine and Difficult Diseases Institute,Second Xiangya Hospital, Central South University, Changsha, 410011 China; 3 Department of Cardiovascular Medicine, The Second Xiangya Hospital, Central South University, Changsha, 410011 Hunan China

**Keywords:** Waist circumference, Type-2 diabetes mellitus, All-cause mortality, Major adverse cardiovascular events

## Abstract

**Background:**

Although studies have shown that waist circumference (WC) is positively associated with an increased risk of cardiovascular diseases among the normal population, few studies have investigated WC in patients with type-2 diabetes mellitus (T2DM).

**Methods:**

This was a post hoc analysis of the Action to Control Cardiovascular Risk in Diabetes (ACCORD) study. The Cox proportional hazards models was used to investigate the relationship between WC and major adverse cardiovascular events (MACEs) in T2DM patients with cardiovascular disease (CVD) or high risk factors of CVD.

**Results:**

A total of 10,251 T2DM patients (6299 men [61.4%], 3952 women [38.6%]) were included in our analysis. The mean age was 64.0 ± 7.53 years. After a mean follow-up at 9.2 ± 2.4 years later, 1804 patients (event rate of 23 per 1000 person-years) had developed MACEs. MACEs rates in men and women were 18.0 and 26.0 events per 1000 person-years, respectively. After multivariable adjustment, each increase in WC of 1 SD increased the risk of MACEs (HR: 1.10, 95% CI 1.04–1.17; P < 0.01) in men, with a non-significant increase in MACEs (HR: 1.04, 95% CI 0.95–1.13; P = 0.40) in women. Compared with those in the first quartile of WC, male patients in the fourth quartile of WC had a hazard ratio (HR) of 1.24 (95% CI 1.05–1.46) for MACEs; female patients in the fourth quartile of WC had an HR of 1.22 (95% CI 0.96–1.56) for MACEs.

**Conclusions:**

Higher WC is associated with increased risks of MACEs in male but not female T2DM patients.

*Trial registration* URL: http://www.clinicaltrials.gov. Unique identifier: NCT00000620)

## Background

Obesity has become a major health problem worldwide and affects almost all of the major cardiovascular risk factors including hypertension, type-2 diabetes mellitus (T2DM), and hyperlipidemia [1–5]. Although obesity is associated with an increased risk of cardiovascular diseases (CVDs) and CVD-related risk factors [3], relevant epidemiologic studies have demonstrated obese or overweight T2DM patients may have better clinical outcomes compared with those at a normal weight [6, 7]. Relevant studies have indicated that this phenomenon is partly attributed to the fact that body mass index (BMI) is an imperfect measure of obesity; additionally, BMI does not reflect body fat distribution and does not discriminate lean body mass from visceral adipose tissue [8, 9]. Our previous study has shown that excess visceral adipose tissue may be detrimental to health, whereas lean body mass may be beneficial to health [10]. Compared with BMI, waist circumference (WC) is a more effective surrogate measure of body fat distribution than BMI and strongly correlates with visceral adipose tissue [11, 12]. Visceral adipose contributes significantly to inflammatory and metabolic complications of obesity [13–16]. However, visceral adipose continuously changes with age, and these changes are different between male and female patients [17]. Men start to lose lean mass after age 50; women show a similar decline in lean mass but gain greater fat mass [18]. Sex differences may exist regarding the relationship between WC and the risk of CVD [19]. Furthermore, for a given WC, there are significant sex differences in visceral adipose tissue [20]. Therefore, sex differences may exist between WC and the risk of CVD.

Although studies have shown that WC has been positively associated with CVDs, all-cause mortality, and new-onset T2DM [21–25], it has rarely been studied among patients with T2DM. Sone et al. found—in a relatively small cohort study—that a high WC value alone is not sufficient to raise the risk of CVD events significantly and is not an independent risk factor in Japanese diabetic patients [26]. Previous study found that the risk of developing long-term MACE differed in different populations [27]. Few studies have investigated sex differences in the relationship between WC and CVD in Western populations. Furthermore, research from non-diabetic populations may not apply in patients with T2DM. Patients with T2DM tend to be more obese than patients without T2DM [28]. Previous studies have also found that T2DM affects sex differences in terms of the risk of CVD; nondiabetic women have a lower risk of CVD than that of men of the same age, but this advantage in women was absent in patients with T2DM [29, 30]. Hence, we used data from the Action to Control Cardiovascular Risk in Diabetes (ACCORD) study to assess sex differences in the relationship between WC and the risk of CVD.

## Methods

### Study population

We performed a post hoc analysis of data from the ACCORD trial, which was a randomized study involving 10,251 T2DM patients at a high risk of CVD. The rationale and design of the ACCORD trial have been described previously, and the results have been published [31–34]. The ACCORD trial, conducted at 77 clinical sites in the United States and Canada, is a double 2 × 2 factorial trial designed to test whether intensified control of blood glucose, blood pressure, and lipids could reduce the incidence of CVD in T2DM patients at a high risk of CVD. This study involved T2DM patients with glycosylated hemoglobin (HbA1c) concentrations of 7.5% or more. The included patients were 40–79 years old and had a history of CVD with anatomical evidence of significant atherosclerosis, albuminuria, left ventricular hypertrophy, or at least two risk factors for cardiovascular disease. Intensive control of both blood pressure and lipids also did not reduce CVD. However, the intensive glycaemia intervention was stopped after a mean follow-up of 3.7 years owing to increased mortality in the intensive glycaemia control arm, and all participants were transitioned to the standard glycaemia control intervention. Follow-up continued for the remaining participants in the ACCORD trial.

### Exposure variables

WC was measured, over bare skin, at the smallest point between the tenth rib and the iliac crest. We treated WC as sex-specific quartiles because the relationship between WC and adverse outcomes was not completely linear.

### Study outcomes

The primary outcome measure of this study was major adverse cardiovascular events (MACEs), defined as a composite of nonfatal myocardial infarction, nonfatal stroke, and/or death from cardiovascular causes [33, 35]. The second endpoints were all-cause mortality, cardiac death, non-fatal myocardial infarction (MI), non-fatal stroke. Participants were followed every 2–4 months. At the 4-month intervals, they were asked about relevant medical events. The MACEs were classified by a Working Group of the Morbidity and Mortality subcommittee. The definitions for cardiac death, MI, and stroke were presented in Additional file 1: Table S1.

### Statistical analysis

We presented baseline characteristics of patients across the quartiles as frequencies and percentages for categorical variables and as means and standard deviations (SDs) or median and interquartile ranges for continuous variables, depending on whether datasets were normally distributed (assessed by normal Q–Q plots). We compared categorical variables using Chi-square analysis, and continuous variables were compared by analysis of variance or Mann–Whitney U-tests, according to the distribution type.

We evaluated the relationship between quartiles of WC and our study endpoints by Cox proportional hazard models. To avoid bias of the Cox proportional hazards models, we performed competing risk regression models (modeling sub-distributional hazard ratios) for cardiac death, non-fatal MI, and non-fatal stroke. The competing event, all-cause mortality, as a permanent condition may prevent the occurrence of non-fatal MI, non-fatal stroke; non-cardiac preventing the occurence of cardiac death. We developed various analyses using four models to reveal the association between WC and MACEs, Model 1: unadjusted; Model 2: adjusted for age, treatment group, and race; Model 3: adjusted for age, treatment group (intensive or standard glucose control), race, hypertension, previous heart failure, hyperlipidemia, smoking, previous cardiovascular disease, proteinuria, and depression; Model 4: same as Model 3, but with the addition of HbA1C and glomerular filtration rate. The proportional hazard assumption was examined and confirmed by graphical methods via the scaled Schoenfeld residuals.

To account for WC as a continuous variable, we constructed a Cox proportional-hazards regression model adjusting for Model 4, in which WC was entered to calculate the HR for MACEs per an increase in WC of 1 SD [36]. We further used restricted cubic splines with four knots at the 5th, 35th, 65th, and 95th centiles to flexibly model the association of WC with the logarithm of the relative risk of MACEs adjusted for model 4 where the mean WC value served as the Ref. [10].

Interaction and stratified analyses were performed according to age (< 60, ≥ 60 years), race, treatment groups, current smoking status, as well as the presence/absence of previous cardiovascular disease, hypertension, and/or hyperlipidemia. We also performed several sensitivity analyses to test the relationship between WC and MACE by excluding participates with age > 75 years or BMI < 18.5 kg/m^2^. We also excluded participants who had follow-up time of less than 2 years because these patients might have unknown diseases. We performed all of the analyses using Stata 15.1 (StataCorp) and R Version 3.4.3 (R Foundation for Statistical Computing, Vienna, Austria).

## Results

### Baseline characteristics of included T2DM patients

Among the total 10,251 T2DM patients, the mean age was 64.0 ± 7.53 years and the majority (61.4%) of the patients were men, as only 3952 (38.6%) were women. After a mean follow-up at 9.2 ± 2.4 years later, 1804 patients (event rate of 23 per 1000 person-years) had developed MACEs. Female patients were more likely to have depression and to have higher cholesterol and high-density lipoprotein levels. MACEs rates in men and women were 18.0 and 26.0 events per 1000 person-years, respectively. The detailed baseline characteristics of the patients with T2DM included in the study population are provided in Table 1.

**Table 1:** Baseline characteristics of included T2DM patients

	Total	Men	Women
n	10,251	6299	3952
WC (cm; mean ± SD)	107 ± 13.6	108 ± 13.3	104 ± 13.8
WC quartiles			
1	90 ± 6.2	92 ± 5.2	86 ± 6
2	102 ± 3.3	103 ± 2.5	99 ± 2.8
3	111 ± 3.3	112 ± 2.8	109 ± 2.9
4	124 ± 6.9	126 ± 7.0	122 ± 6
Central adiposity (%)^a^	7534 (73.5)	4081 (64.8)	3453 (87.3)
BMI (kg/m^2^; mean ± SD)		31.6 ± 5.05	33.2 ± 5.78
Age (year; mean ± SD)	62.8 ± 6.65	63.0 ± 6.81	62.6 ± 6.38
Race (%)			
White	6393 (62.4)	4234 (67.2)	2159 (54.6)
Non-white	3858 (37.6)	2065 (32.8)	1793 (45.4)
Median duration of diabetes (year; mean ± SD)	10.8 ± 7.6	10.8 ± 7.60	10.8 ± 7.6
Hypertension (%)	7726 (75.4)	4630 (73.5)	3096 (78.3)
Hyperlipidemia (%)	7165 (69.9)	4452 (70.7)	2713 (68.6)
Previous cardiovascular events	3609 (35.2)	2586 (41.1)	1023 (25.9)
Current smoker (%)	1429 (13.9)	961 (15.3)	468 (11.8)
Previous heart failure (%)	494 (4.80)	309 (4.90)	185 (4.70)
Proteinuria (%)	2035 (19.9)	1286 (20.4)	749 (19.0)
Depression (%)	2421 (23.6)	1261 (20)	1160 (29.4)
Heart rate (bpm)	72.7 ± 11.8	71.8 ± 11.9	74.1 ± 11.3
SBP (mmHg, mean ± SD)	136 ± 17.1	135.6 ± 16.6	137.5 ± 17.9
DBP (mmHg, mean ± SD)	74.9 ± 10.7	74.6 ± 10.7	75.3 ± 10.6
Glycated hemoglobin (%, mean ± SD)	8.30 ± 1.06	8.27 ± 1.04	8.34 ± 1.07
GFR (mL/min, mean ± SD)	91.0 ± 27.2	90.9 ± 22.9	91.2 ± 32.8
FPG (mg/dL, mean ± SD)	175 ± 56.2	176 ± 57.0	174 ± 54.8
Cholesterol (mg/dL, mean ± SD)			
Total	183 ± 41.9	177 ± 40.1	194 ± 42.4
Low-density lipoprotein	104 ± 33.9	101 ± 32.4	111 ± 35.3
High-density lipoprotein	41.9 ± 11.6	38.6 ± 9.65	47.1 ± 12.6
Medications (%)			
Insulin	1143 (11.2)	672 (10.7)	471 (11.9)
Metformin	6554 (63.9)	4099 (65.1)	2455 (62.1)
Sulfonylurea	5474 (53.4)	3518 (50.5)	1956 (49.5)
Thiazolidinedione	2258 (22.0)	1411 (22.4)	847 (21.4)
Beta-blockers	3079 (30.1)	2009 (32.0)	1070 (27.1)
ACEI	5568 (54.5)	3608 (57.4)	1960 (49.7)
Statin	6500 (63.7)	4185 (66.0)	2315 (58.9)
Aspirin	5579 (54.7)	3639 (58.0)	1940 (49.4)

*WC* waist circumference, *BMI* body mass index, *SBP* systolic blood pressure, *DBP* diastolic blood pressure, *GFR* glomerular filtration rate, *FPG* fasting plasma glucose, *ACEI* angiotensin-converting enzyme inhibitors

^a^Central adiposity was defined as a WC of ≥ 102 cm in men and ≥ 88 cm in women

### Quartiles of WC and MACEs

Table 2 shows the association between WC and MACEs in the included T2DM patients. The risk of MACEs increased in males with each higher quartile of WC in the first four models. Compared with those in the first quartile of WC, men in the fourth quartile of WC had an HR of 1.24 (Model 4, 95% CI 1.05–1.46, P = 0.02) for MACEs. women in the fourth quartile of WC had an HR of 1.22 (95% CI 0.96–1.56, P = 0.401, Model 4) for MACEs. Higher WC was not associated with a higher risk of MACE in female T2DM patients.

**Table 2:** Quartiles of WC and MACEs

WC quartile	Event rate^a^	Hazard ratio (95% CI)			
		Model 1	Model 2	Model 3	Model 4
Men					
1	23.1	Ref	Ref	Ref	Ref
2	24.7	1.07 (0.91–1.26)	1.04 (0.89–1.23)	1.03 (0.88–1.22)	1.04 (0.89–1.24)
3	26.9	1.19 (1.00–1.37)	1.14 (0.97–1.34)	1.12 (0.95–1.32)	1.13 (0.96–1.33)
4	29.5	1.28 (1.09–1.50)	1.28 (1.09–1.50)	1.23 (1.04–1.45)	1.24 (1.05–1.46)
P value for trend		< 0.01	< 0.01	0.02	0.02
Women					
1	16.5	Ref	Ref	Ref	Ref
2	18.4	1.11 (0.88–1.41)	1.12 (0.88–1.42)	1.18 (0.92–1.50)	1.19 (0.93–1.52)
3	16.6	1.01 (0.798–1.29)	1.02 (0.80–1.31)	1.00 (0.78–1.28)	0.994 (0.78–1.27)
4	20.6	1.24 (0.99–1.58)	1.29 (1.01–1.64)	1.24 (0.97–1.58)	1.22 (0.96–1.56)
P value for trend		0.17	0.12	0.18	0.40

^a^Per 1000 person-years. Model 1: unadjusted; Model 2: adjusted for age, treatment group (intensive or standard glucose control), race; Model 3 adjusted for age, treatment group, race, hypertension, previous heart failure, hyperlipidemia, smoking, previous cardiovascular disease, proteinuria, depression; model 4: model 3 in addition to HbA1C, glomerular filtration rate; Ref: reference

### WC as a continuous variable and MACEs

When we used WC as a continuous covariate in the fully adjusted Cox proportional hazards model (Model 4), each increase in WC of 1 SD increased the risk of MACEs (HR: 1.10, 95% CI 1.04–1.17; P < 0.01) in men, with a non-significant increase in MACEs (HR: 1.04, 95% CI 0.95–1.13; P = 0.40) in women. WC was not associated with MACEs among female T2DM patients.

**Fig. 1:**
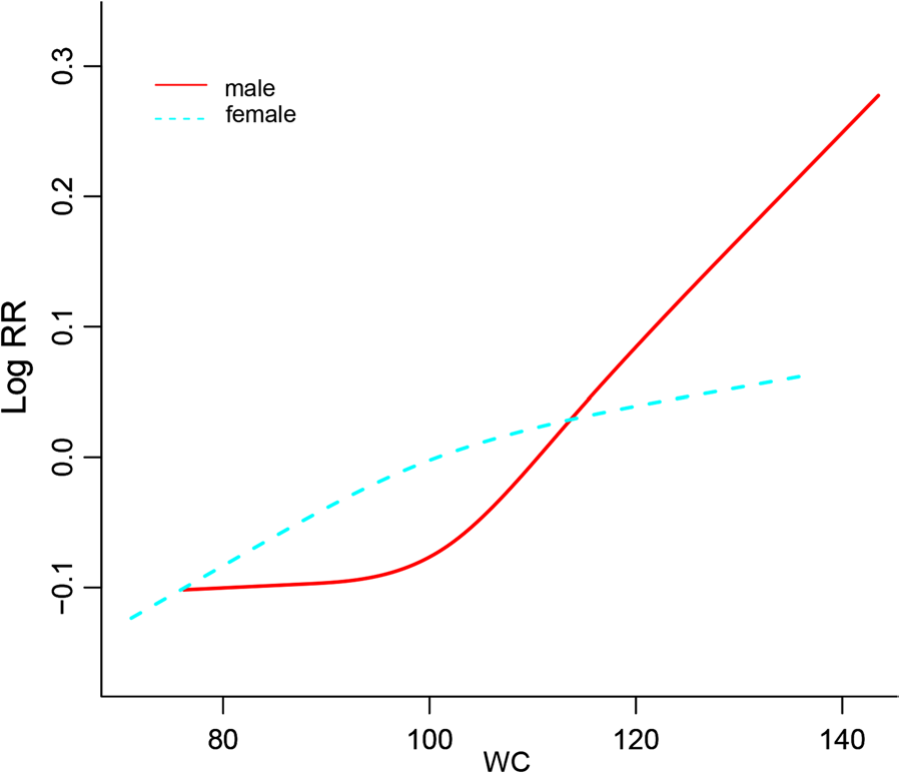
Smooth spline curves of WC for the estimation of risk of MACEs after adjusting multivariate rates. *WC* waist circumference

We next used restricted cubic splines to flexibly model and visualize the relation of WC with MACEs both in men and women. The risk of MACEs increased rapidly with increasing WC which suggested that a extremely high WC might be associated with MACEs in men; However, the curve was relatively flat in women which suggested that women with higher WC only slightly increased the risk of MACEs (Fig. 1).

### Second endpoints

Higher WC was positively associated with all-cause mortality in both men and women with T2DM. Compared with those in the first quartile of WC, men in the fourth quartile of WC had an HR of 1.49 (Model 4, 95% CI 1.27–1.83, P for trend < 0.01, Model 4) for MACEs. women patients in the fourth quartile of WC had an HR of 1.58 (95% CI 1.25–2.00, P for trend < 0.01, Model 4) for MACEs. Men with higher quartile of WC had increased risk of cardiac death; however women with quartile of WC had higher risk of non-fatal stroke (Table 3).

**Table 3:** Quartiles of WC and second endpoints

WC quartile	Hazard ratio (95% CI)			
	Cardiac death	Non-fatal MI	Non-fatal stroke	All-cause Mortality
Men				
1	Ref	Ref	Ref	Ref
2	0.96 (0.72–1.26)	1.02 (0.81–1.27)	1.67 (1.22–2.29)	1.03 (0.88–1.21)
3	1.09 (0.83–1.43)	1.07 (0.86–1.34)	1.38 (0.99–1.91)	1.18 (1.00–1.38)
4	1.66 (1.28–2.16)	1.01 (0.81–1.50)	1.14 (0.81–1.62)	1.49 (1.27–1.83)
P value for trend	< 0.01	0.84	0.95	< 0.01
Women				
1	Ref	Ref	Ref	Ref
2	1.18 (0.78–1.80)	0.92 (0.66–1.29)	1.79 (1.08–2.94)	1.23 (0.97–1.56)
3	0.96 (0.62–1.50)	0.78 (0.56–1.11)	1.62 (1.62–2.67)	1.11 (0.87–1.41)
4	1.17 (0.77–1.79)	1.01 (0.73–1.40)	1.88 (1.14–3.09)	1.58 (1.25–2.00)
P value for trend	0.66	0.92	< 0.01	< 0.01

Model 4: adjusted for age, treatment group (intensive or standard glucose control), race, hypertension, previous heart failure, hyperlipidemia, smoking, previous cardiovascular disease, proteinuria, depression, HbA1C, glomerular filtration rate; Ref: reference

### Interaction and sensitivity analyses

**Fig. 2:**
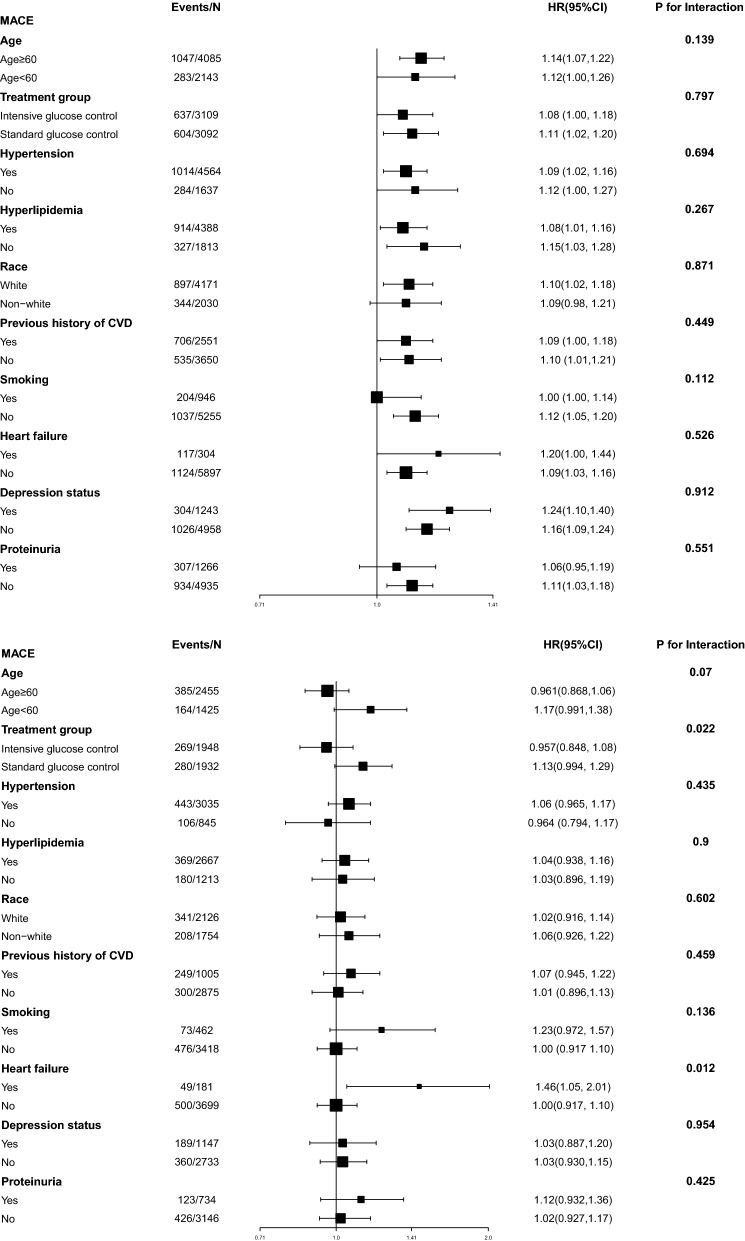
The HR per SD increase in WC for MACEs. **a** Data for male T2DM patients are shown. **b** Data for female T2DM patients are shown. Each stratification was adjusted for all factors in Model 4, except for the stratification factor itself. *MACEs* major adverse cardiovascular events

Figure 2 shows the association between WC and MACEs in the different subgroups. We did not find interactions among WC and age, treatment group, hypertension, hyperlipidemia, race, previous history of CVD, smoking, depression status, or proteinuria among male T2DM patients. However, we did find heart failure moderated the association between WC and MACE in females which suggested a stronger effect of WC on MACE among those women with heart failure than those without.

In order to further verify the association between WC and MACEs, we excluded MACE events in patients > 75 years old, or with a BMI < 18.5 kg/m^2^ or follow-up less than 2 years. After these exclusions, our sensitivity analyses showed that the above results regarding WC and MACEs remained robust among both male and female T2DM patients (Additional file 1: Table S2).

## Discussion

In our post hoc analysis involving patients with a mean 10-year history of T2DM who had a high risk of CVD, we found a significant sex difference in the relationship between WC and cardiovascular events. WC had a strong positive association with CVD in male but not female patients.

Previous studies have found that patients with higher WC have a higher risk of adverse events including new-onset T2DM, all-cause mortality, and cardiovascular events [37–39]. However, few studies have investigated the relationship between WC and MACEs in T2DM patients. Patients with T2DM tend to be more obese and have more cardiovascular risk factors—including hypertension and hyperlipidemia—compared with patients without T2DM. Similarly, patients with higher WC also have more cardiovascular risk factors. However, the relationship between WC and cardiovascular events has remained uncertain in this population. Sone et al. studied the relationship between WC and cardiovascular disease in Japanese T2DM patients without CVD in a small cohort study; they found that WC was not a sufficient predictor of MACEs [26]. In the present study, we found that WC was not a sufficient predictor of MACEs in female T2DM patients. These findings challenge our view of the relationship between WC and cardiovascular disease in T2DM patients. Importantly, however, the previously established relationship between WC and CVD has derived from normal populations without T2DM patients. T2DM patients tend to be more obese and have more risk factors such as hypertension and hyperlipidemia compared with patients without T2DM. Therefore, the results regarding the relationship between WC and cardiovascular diseases from normal populations may not be applicable to patients with T2DM.

Our present study found that WC may not be a good predictor of cardiovascular events in female T2DM patients. This phenomenon may be attributed to sex differences in body fat and fat distribution. Females tend to have more abdominal fat compared with that of men [40, 41]. Furthermore, women have 10% higher body fat compared to men with the same BMI [42, 43]. Aging increases adiposity in both women and men; furthermore, women are characterized by having a higher percent of fat. Relevant studies found that female hearts are more dependent of fatty acids for energy production and uptake more fatty acids compared with male hearts which may explain the advantage of female patients with more fat and higher fatty acids [44, 45]. In our study, female patients have lower risks of MACEs, though female patients with T2DM have higher lipid levels. Epidemiologic studies also found that this paradox seems more prominent in female patients [46, 47]. These may be the reason why WC is a good indicator of fat content rather than the risks of CVD in female patients with T2DM.

The present study has several limitations. First, we calculated WC on the basis of WC at the inception of the study, and we did not reevaluate WC during the follow-up. Beleigoli et al., however, found that changes in WC were not significantly associated with mortality [48]. Our previous study also found that the average change in weight across the entire study was 1.80%, representing 0.448 kg/m^2^; therefore, any change in WC is likely relatively small [49]. Second, all included patients were Caucasians, and these results may not apply to other populations (e.g., an Asian population) that exhibit different habits. Third, the period of follow-up was relatively short. Fourth, there are about 1300 events in men and 550 events in women; consequentially, the uncertainty of the estimates for the associations in women is higher than for men.

## Conclusion

In T2DM patients, a positive relationship between WC and MACEs is observed in male but not female patients. Higher WC in male patients is significantly associated with higher risk of MACEs. However, a high WC alone is not sufficient to raise the risks of MACEs in female patients with T2DM.

## Supplementary information

**Additional file 1: Table S1.** The definitions for cardiac death, MI, and stroke. **Table S2.** Sensitivity analysis.

## Data Availability

Data are available from the Biologic Specimen and Data Repository Information Coordinating Center (BioLINCC).

## Abbreviations

T2DM: Type-2 diabetes mellitus
CVD: Cardiovascular disease
ACCORD: The Action to Control Cardiovascular Risk in Diabetes
WC: Waist circumference
BMI: Body mass index
SD: Standard deviation
MACE: Major adverse cardiovascular events
SBP: Systolic blood pressure
DBP: Diastolic blood pressure
GFR: Glomerular filtration rate
ACEI: Angiotensin-converting enzyme inhibitors
FPG: Fasting plasma glucose
HbA1C: Glycosylated hemoglobin
