# Supplementary material for: Waist circumference is associated with major adverse cardiovascular events in male but not female patients with type-2 diabetes mellitus
Source: Cardiovasc Diabetol. 2020 Mar 25;19:39. doi: 10.1186/s12933-020-01007-6 (PMC7093979; doi:10.1186/s12933-020-01007-6)
Supplement: Supplementary file 1 — Additional file 1: Table S1. The definitions for cardiac death, MI, and stroke. Table S2. Sensitivity analysis. [file 12933_2020_1007_MOESM1_ESM.docx]

Table S1: The definitions for cardiac death, MI, and stroke

| **Cardiovascular Death** |
| --- |
| 1.Unexpected death: Unexpected death presumed to be due to ischemic cardiovascular  disease, occurring within 24 hours of the onset of symptoms without confirmation of  cardiovascular disease, and without clinical or post mortem evidence of other etiology.   1. Fatal Myocardial infarction (MI): death within 7 days of the onset of documented MI 2. Congestive heart failure (CHF): death due to clinical, radiological or postmortem evidence of CHF without clinical or postmortem evidence of an acute ischemic event(cardiogenic shock to be included). 3. Death after invasive cardiovascular interventions: death associated with the intervention 4. Documented arrhythmia: death due to bradyarrhythmias or tachyarrhythmias not associated with an acute cardiac ischemic event. 5. Death following non-cardiovascular surgery 6. Stroke: death due to stroke occurring within 7 days of the signs and symptoms of a stroke 7. Other cardiovascular diseases: death due to other vascular diseases including pulmonary emboli and abdominal aortic aneurysm rupture. 8. Presumed cardiovascular death: Suspicion of cardiovascular death with supportingclinical evidence that may not fulfill criteria otherwise stated. |
| **MI** |
| The definitions for MI are presented below. If necessary for a definition, prolonged ischemic symptoms must last 20 minutes, and the cardiac enzymes of interest are Troponin T or I and/or serum CK-MB mass. Silent MIs will be identified by the ACCORD ECG Reading  1.Q-wave MI,2.Non Q-wave MI,3.Silent (unrecognized) MI,4.Probable non Q-wave MI,5.MI after cardiovascular invasive interventions,6.MI after coronary bypass graft surgery,7.MI after non-cardiovascular surgery |
| **Stroke** |
| 1.Definite ischemic stroke,2.Definite primary intracerebral hemorrhage,3.Subarachnoid hemorrhage,4.Stroke of unknown type etiology,5.Non-fatal stroke after cardiovascular invasive interventions6.Non-fatal stroke post non-cardiovascular surgery |

Table S2 Sensitivity analysis

| WC quartile | Hazard ratio(95%CI) | | |
| --- | --- | --- | --- |
|  | **#** | **$** | **§** |
| Men |  |  |  |
| 1 | Ref | Ref | Ref |
| 2 | 1.09(0.92–1.28) | 1.08(0.91–1.28) | 1.03(0.85–1.24) |
| 3 | 1.13(0.96–1.33) | 1.10(0.93–1.31) | 1.04(0.86–1.25) |
| 4 | 1.27(1.08–1.50) | 1.23(1.04–1.46) | 1.28(1.06–1.54) |
| P value for trend | <0.01 | <0.01 | <0.01 |
| Women |  |  |  |
| 1 | Ref | Ref | Ref |
| 2 | 1.14(0.88–1.47) | 1.19(0.93–1.52) | 1.16(0.87–1.54) |
| 3 | 0.98(0.75–1.27) | 0.99(0.77–1.27) | 0.99(0.74–1.31) |
| 4 | 1.23(0.95–1.58) | 1.22(0.96–1.56) | 1.37(0.99–1.81) |
| P value for trend | 0.28 | 0.28 | 0.06 |

Model 4:adjusted for age, treatment group, race, hypertension, previous heart failure, hyperlipidemia, smoking, previous cardiovascular disease, proteinuria, depression, HbA1C, glomerular filtration rate; Ref: reference.

**#** excluding participates with age >75 years

**$** excluding participates withBMI <18.5 kg/m^2^.

**§**participants who had follow-up time of less than two years
